# Exploring Post‐Retrieval Strategies to Reduce Drug Craving in Methamphetamine Use Disorders

**DOI:** 10.1111/adb.70049

**Published:** 2025-06-19

**Authors:** Junjiao Li, Yuanyuan Dong, Wei Chen, Jian Wang, Xifu Zheng

**Affiliations:** ^1^ College of Teacher Education Guangdong University of Education Guangzhou China; ^2^ School of Psychology South China Normal University Guangzhou China; ^3^ Center for Studies of Psychological Application South China Normal University Guangzhou China; ^4^ Guangdong Key Laboratory of Mental Health and Cognitive Science Guangzhou China; ^5^ The Affiliated Brain Hospital of Guangzhou Medical University Guangzhou China; ^6^ Shenzhen Compulsory Isolation and Drug Rehabilitation Center Shenzhen China

**Keywords:** cognitive task, craving, methamphetamine use disorder, post‐retrieval intervention, retrieval–extinction

## Abstract

Post‐retrieval interventions based on memory reconsolidation have shown promise in reducing addiction‐related memories. However, research on methamphetamine (MA) use, particularly in humans, remains limited. This study aimed to evaluate the efficacy of a post‐retrieval intervention paradigm in managing methamphetamine use disorder (MUD) with 46 individuals from a compulsory drug rehabilitation centre. A single‐blind design was employed, with participants randomly assigned to one of three groups: (1) retrieval–no intervention, (2) retrieval–extinction and (3) retrieval–cognitive task. The study involved baseline testing, followed by memory retrieval using MA cues, and one of the three interventions during the memory reconsolidation window. The interventions were as follows: (1) no further intervention after retrieval, (2) extinction training and (3) playing Tetris after memory reactivation. Relapse was assessed through physiological and psychological indicators, with a focus on both spontaneous and cue‐induced relapse of MUD memory. The results showed that both retrieval–extinction and retrieval–cognitive task showed benefits in reducing cravings and preventing relapse in MUD compared to retrieval alone. Physiological and psychological indicators of MA memory relapse showed weak correlation and differed across several dimensions. These findings suggest new strategies for MUD intervention and provide valuable insights for clinical treatment. Limitations of the study are also discussed.

## Graphical abstract

To assess post‐retrieval interventions for MUD, a single‐blind study randomised participants into three groups: (1) retrieval‐no intervention (R), (2) retrieval‐extinction (R‐E) and (3) retrieval‐cognitive task (R‐CT). Results demonstrated that G2 (R‐E) significantly reduced drug cravings compared to G1, with sustained lower cravings during spontaneous recovery at the 1‐month follow‐up. G3 (R‐CT) also exhibited craving suppression in subsequent tests.

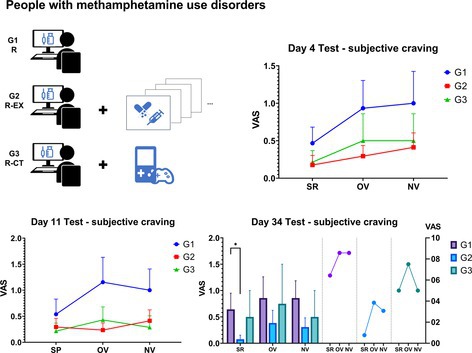

## 1 Introduction

Methamphetamine (MA) is a highly addictive central nervous system stimulant. Methamphetamine use disorder (MUD) is identified in clinical practice by significant psychological and psychiatric disturbances due to substance addiction or misuse, with a hallmark being the inability to regulate compulsive drug consumption [1]. Drug addiction disrupts the brain's natural reward circuits, hijacking pathways responsible for physiological needs, thereby prioritizing drug use over innate necessities [2]. Despite the critical need for effective treatment options, there are currently no FDA‐approved medications for MUD. Current treatments primarily involve psychological and behavioural therapy. However, their limited effectiveness—despite various supportive psychosocial interventions—underscores the need for more effective therapeutic strategies. A central issue in MUD treatment is relapse, which is often driven by persistent drug‐related memories. These memories can endure long after abstinence and are typically resistant to erasure through traditional extinction methods [3], which only establish conditioned stimulus (CS)–no unconditioned stimulus (US) learning to inhibit the original CS‐US association, leaving the underlying drug‐related memory intact.

Craving, defined as a strong impulse to obtain and use the drug, can be triggered even after extended abstinence by exposure to stress, drug cues or familiar environments [4]. Addiction is increasingly recognized as a disorder of learning and memory, where both Pavlovian and instrumental conditioning are co‐opted by drug use to reinforce drug‐seeking behaviour [5].

In recent years, the retrieval–extinction paradigm, grounded in memory reconsolidation theory, has emerged as a more effective approach for reducing cravings and preventing relapse. According to the reconsolidation theory, when the original memory is reactivated, it becomes unstable (destabilized), providing a window during which extinction training can interfere with memory reconsolidation, potentially modifying or even deleting the original memory [6, 7, 8]. Research across various drug addictions, including cocaine, heroin and morphine, has demonstrated the effectiveness of retrieval–extinction in suppressing relapse and craving. For instance, retrieval–extinction and methadone‐initiated memory reconsolidation significantly reduced drug‐seeking behaviours in both animal models and humans addicted to morphine and heroin [9, 10]. Similarly, retrieval–extinction reduced cocaine relapse, with US retrieval–extinction proving superior to CS retrieval–extinction [11, 12]. In alcohol and nicotine addiction, post‐retrieval interventions have also shown effectiveness, with studies exploring neural mechanisms involved in relapse prevention [13, 14, 15, 16]. Various post‐retrieval interventions, including behavioural and pharmacological methods, have been studied, mainly in alcohol addiction. Techniques like extinction training, counterconditioning and cognitive tasks (e.g., working memory training and cognitive reappraisal) have effectively reduced alcohol‐related cravings and behaviours [13, 17, 18, 19]. Additionally, pharmacological approaches, such as N_2_O and ketamine, show promise in disrupting alcohol memory reconsolidation and reducing drinking [20, 21].

However, research on new drugs like methamphetamine remains limited, highlighting a significant gap in the literature. Initial studies in animal models have shown that retrieval–extinction can prevent the reinstatement of MA addiction memories, with alterations in basolateral amygdala (BLA) neuron activation potentially being a key neural mechanism for inhibiting relapse [22]. To date, only one study has applied this method to humans, using virtual reality (VR) to reduce methamphetamine craving [23]. Hence, although post‐retrieval interventions have shown preliminary success in addiction memory extinction, research on methamphetamine is scarce, particularly in human studies. Findings from traditional drug studies may not be directly applicable to methamphetamine addiction.

To address these gaps, the present study conducts a retrieval intervention experiment in individuals undergoing detoxification for MUD, while comparing three behavioural procedures. The phrase “MUD memory relapse” describes the reemergence of conditioned responses (e.g., craving and physiological arousal) triggered by reactivated drug‐associated memories. This process parallels fear memory relapse, where extinguished memories regain salience under specific conditions (e.g., stress and reexposure to cues), thereby increasing relapse risk [24, 25]. We compared post‐retrieval extinction, a visuospatial working memory task, and a no‐intervention control on cue reactivity in MUD. Our hypothesis is that post‐retrieval interventions could effectively decrease both the physiological and psychological responses linked to MUD and prevent relapse.

## 2 Materials and Methods

### 2.1 Participants

The study was approved by the Ethics Committee at South China Normal University (Approval Number: SCNU‐PSY‐2021‐329) and received cooperation from the detox centre. Prior to the experiment, a survey and preliminary screening were conducted among all individuals at the detox centre. Participants, aged 18–60, had to meet DSM‐V criteria for substance use disorder and primarily use methamphetamine before entering the detox centre. Individuals with other substance or alcohol use disorders, those with physical disorders or psychiatric conditions requiring treatment or those unwilling to participate in the experiment or follow‐up were excluded. To protect privacy, only in‐centre numbers were recorded, not names or identification numbers. Before starting the experiment, participants were informed that no invasive methods such as drugs or devices would be used and that they could withdraw at any time if they felt uncomfortable. Participation or withdrawal from the experiment and the results would not affect their subsequent treatment or duration of detoxification in the centre. Participants signed consent forms and were given a small gift upon completion of the experiment. The formal experiment involved 46 participants (two females, aged 36.93 ± 7.59), with MA (97.8%) and 3,4‐methylenedioxy‐methamphetamine (MDMA, 2.2%) addictions. Participants were randomly assigned to one of three groups, each with a different intervention strategy: retrieval–no intervention (G1), retrieval–extinction (G2) and retrieval–cognitive task (G3). Participant details are in Table 1.

**TABLE 1:** Participants' information.

Variable	Mean (SD)			*F* or *X* ^2^	*p*
	G1: R (*n* = 15)	G2: R‐E (*n* = 17)	G3: R‐CT (*n* = 14)		
Age (years)	35.33 (6.510)	35.18 (7.796)	40.79 (7.475)	2.80	0.072
Sex (% male)	13 (86.67%)	17 (100%)	14 (100%)	4.32	0.120
Educational background				5.42	0.294
Primary school or below	2 (13.33%)	4 (23.53%)	2 (14.28%)		
Junior high school	5 (33.33%)	8 (47.06%)	6 (42.86%)		
High school	5 (33.34%)	4 (23.53%)	6 (42.86%)		
College	3 (20.00%)	1 (5.88%)	0 (0%)		
Marital status				6.14	0.809
Single	4 (26.67%)	8 (47.06%)	4 (28.57%)		
Married	0 (0%)	0 (0%)	0 (0%)		
Divorced	10 (66.66%)	5 (29.41%)	9 (64.29%)		
Not specified	1 (6.67%)	4 (23.53%)	1 (7.14%)		
Type of drug used					
MA	15 (100%)	16 (94.12%)	14 (100%)		
MDMA	0 (0%)	1 (5.88%)	0 (0%)		
Total duration of use (years)	3.77 (2.85)	6.55 (5.18)	8.54 (6.68)	3.206	0.050
Typical single dose (grammes/pills)	0.49 (0.29)	0.60 (0.92)	0.19(0.12)	2.031	0.144
Questionnaire					
BDI	15.53 (6.24)	12.59 (8.49)	15.14 (11.20)	0.533	0.591
STAI‐T	45.8 (8.26)	44.82 (7.66)	45.13 (7.26)	0.111	0.895

*Note:* Mean values and standard deviations are presented per group.

Abbreviations: BDI, Beck Depression Inventory; MA, methamphetamine; MDMA, 3,4‐methylenedioxy‐methamphetamine; STAI‐T, State–Trait Anxiety Inventory‐Trait Version.

### 2.2 Materials

The subjective questionnaires included the Beck Depression Inventory (BDI) and the State–Trait Anxiety Inventory–Trait Version (STAI‐T).

Experimental materials were categorized into three types: photo cues, video cues (drug‐related and neutral) and paraphernalia cues. Original stimuli were sourced from the internet and standardized for evaluation. Drug‐related photo cues included 120 photos of drugs and paraphernalia, the consumption process and consumption locations. Drug‐related video cues were extracted from international addiction‐related films and documentaries, edited into 5‐min segments, totalling six videos. Simulated paraphernalia and drugs (models, five items) were sourced from the detox centre, including models of MA, MDMA and related items. Neutral videos were background music‐free videos (see the Supplementary Material SM 1‐2, Data S1).

Materials were evaluated by MUD individuals at the detox centre (nonexperimental participants), who were shown the photos or videos and asked to imagine themselves as observers or participants in the scenes. All evaluation settings were consistent. Materials were rated on a 9‐point scale for ‘pleasure’, ‘arousal’ and ‘craving’. Based on the appraisal results, 55 drug‐related photos, 3 drug‐related videos, and 1 neutral video were selected for the formal experiment. Detailed appraisal data are in the Supplementary Materials (SM 3, Data S1). The video used to retrieval drug memory in Day 2 is different from extinction.

### 2.3 Procedure

**FIGURE 1:**
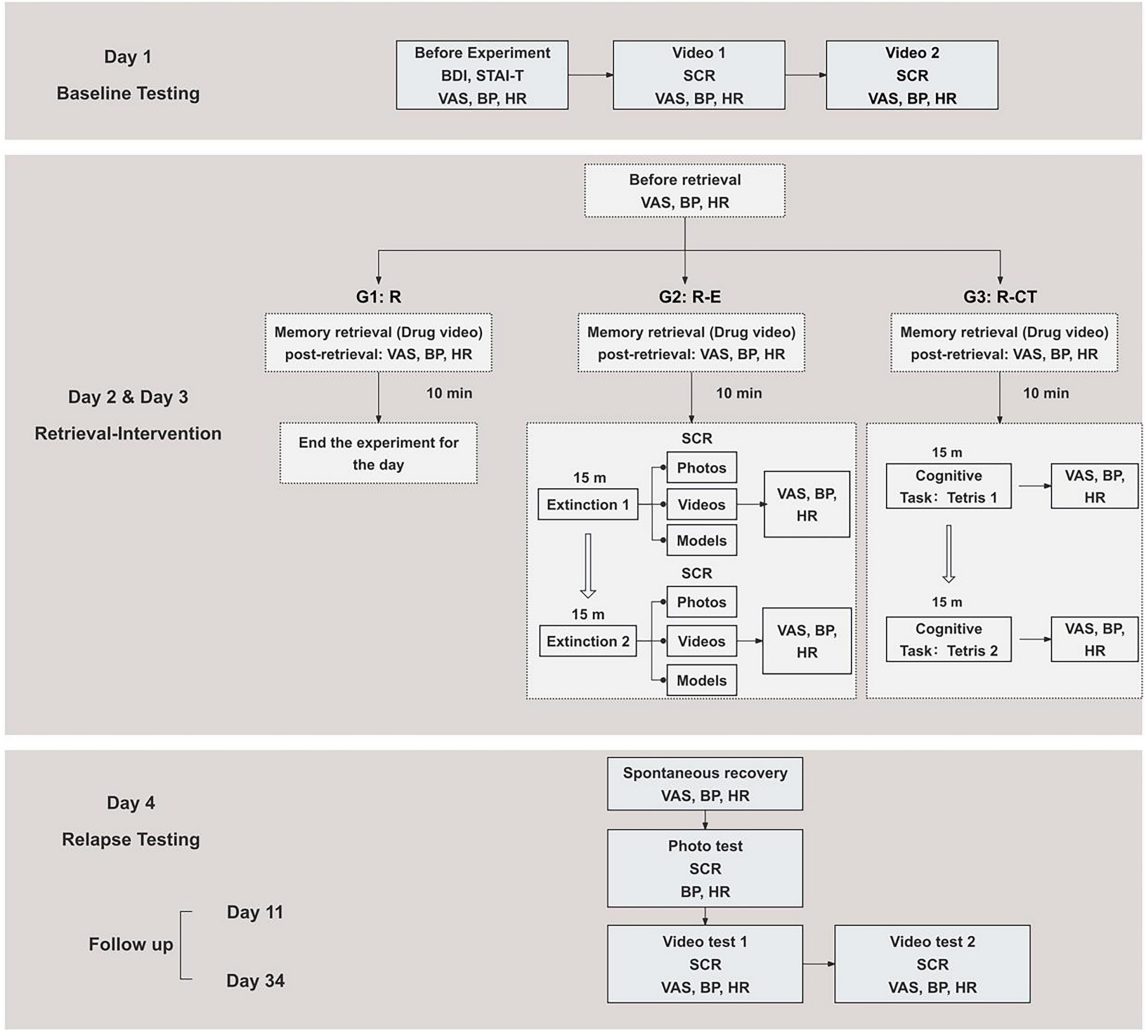
Schematic overview of the experimental design and procedure, consisting of three phases: baseline testing, memory retrieval intervention and relapse testing. After memory reactivation on Day 2, participants were randomly assigned to one of three groups, each with a different intervention strategy: G1 (retrieval–no intervention, R), G2 (retrieval–extinction, R‐E), and G3 (retrieval–cognitive task, R‐CT). The procedure on Day 3 mirrored that of Day 2. Abbreviations: BP, blood pressure; HR, heart rate; m, minutes; SCR, skin conductance response; VAS, Visual Analogue Scale.

The experiment adopts a classic three‐phase retrieval–intervention paradigm, divided into a 4‐day experimental period, followed by a subsequent follow‐up period. The experimental design is illustrated in Figure 1.

#### 2.3.1 Day 1: Baseline Testing

On the first day, participants underwent baseline testing using two 5‐min videos (one neutral and one drug related). Psychological and physiological indicators were measured before and after each video. Participants completed the BDI and STAI‐T scales, followed by data collection on drug craving, heart rate (HR) and blood pressure (BP). Videos were shown in random order with a 10‐min interval, and participants rated their drug craving after each video, with immediate HR and BP measurements taken while skin conductance response (SCR) was continuously recorded.

#### 2.3.2 Days 2 and 3: Retrieval–Intervention

Before memory retrieval, craving levels, HR and BP were measured. Participants then watched a 5‐min MA‐related video to reactivate addiction memories. After the video, measurements were repeated, followed by a 10‐min rest. G1 (R) completed the same task on both days without additional intervention. G2 (R‐E) underwent extinction training, which included viewing MA‐related photos and videos, and interacting with drug models. In photo extinction, a red fixation point “**+**” was shown on the screen for 2000 ms, followed by MA‐related photos (CS) each shown for 5000 ms, totalling 40 CS photos. In video extinction, participants watched an MA‐related video. In model extinction, participants held and observed drug models and paraphernalia, with five different items presented sequentially, each for 5500 ms. Extinction lasted for 15 min, with two sessions totalling 30 min. G3 (R‐CT) engaged in a 30‐min cognitive task, playing Tetris to disrupt memory reconsolidation. Tetris is a type of visuospatial task that involves generating and manipulating mental images, such as rotating mental shapes. It had been shown to effectively reduce intrusive memories within a week of trauma [26] and to reduce traumatic memories through memory reconsolidation [27]. The procedures were identical on the third day for each group. All the photos, videos and models used during the extinction phase were presented in a randomized order to prevent order effects.

#### 2.3.3 Day 4 and Follow‐Up: Relapse Tests

On Day 4, participants were tested for spontaneous recovery and cue‐elicited recurrence using old and novel drug‐related photos and videos, with HR, BP and SCR continuously recorded. Follow‐up tests on Days 11 and 34 replicated Day 4's procedures to assess memory retention in the three groups.

### 2.4 Measurement

#### 2.4.1 Physiological Measurement and Processing

Physiological indicators, including HR, BP and SCR, were primary measures of MUD‐related responses. HR and BP (systolic and diastolic) were recorded at fixed time points using the Yuwell YE660D BP monitor. SCR was measured with the BIOPAC MP36 recorder (BIOPAC Systems Inc., Goleta, CA, United States) and analysed using BIOPAC Student Lab 4.1 software. Two Ag/AgCl electrodes were attached to the ring and index fingers of the left hand with conductive gel. SCR data were sampled at 1000 Hz and processed according to stimulus type. For photo stimuli, peak‐to‐peak (Vpp) values were recorded during the 5000 ms presentation. For video stimuli, maximum amplitude values were recorded at three intervals (0–1, 2–3 and 4–5 min) of the 5‐min video. For paraphernalia stimuli, maximum amplitude values were recorded at three intervals (0–15, 20–35 and 40–55 s) of a 55‐s manipulation period. SCR values below 0.02 μs were set to zero, and remaining values were square root transformed for normalization [28, 29].

#### 2.4.2 Psychological Measurement

Psychological indicators include subjective craving, the primary measure of MUD severity and assessed using the Visual Analogue Scale (VAS). Participants mark their craving intensity on a 0–9 scale, where 0 indicates *no craving* and 9 indicates *extreme craving*.

Although relapse is ultimately confirmed via behavioural outcomes (e.g., drug use), craving and cue‐induced physiological responses serve as clinically validated proxies for relapse risk. These measures reflect the reactivation of drug‐associated memories and conditioned motivational states that precede overt relapse behaviour [25, 30].

### 2.5 Statistical Analyses

Statistical analyses were performed to examine Day 1 baseline and cue validity, Days 2 and 3 intervention processes, and Day 4 relapse and follow‐up assessments. On Days 2 and 3, the retrieval intervention phases were analysed based on the experimental design. G1 only underwent memory retrieval, G2 included extinction and G3 involved a cognitive task where SCR data could not be reliably collected, so only VAS and other physiological data were analysed for G2 and G3.

Data were analysed using JASP 0.16.3 (Intel) and GraphPad Prism 9. Repeated‐measurement analysis of variance (RMANOVA) was used to analyse psychological and physiological indicators for within‐subject and between‐subject factors at each phase. To control for the impact of participant attrition at different follow‐up time points, a linear mixed model (LMM) was employed. A significance level of *p* < 0.05 was used, with *η* ^2^ reported as the effect size estimate.

## 3 Results

### 3.1 Day 1 Baseline Testing and Cue Validity

**FIGURE 2:**
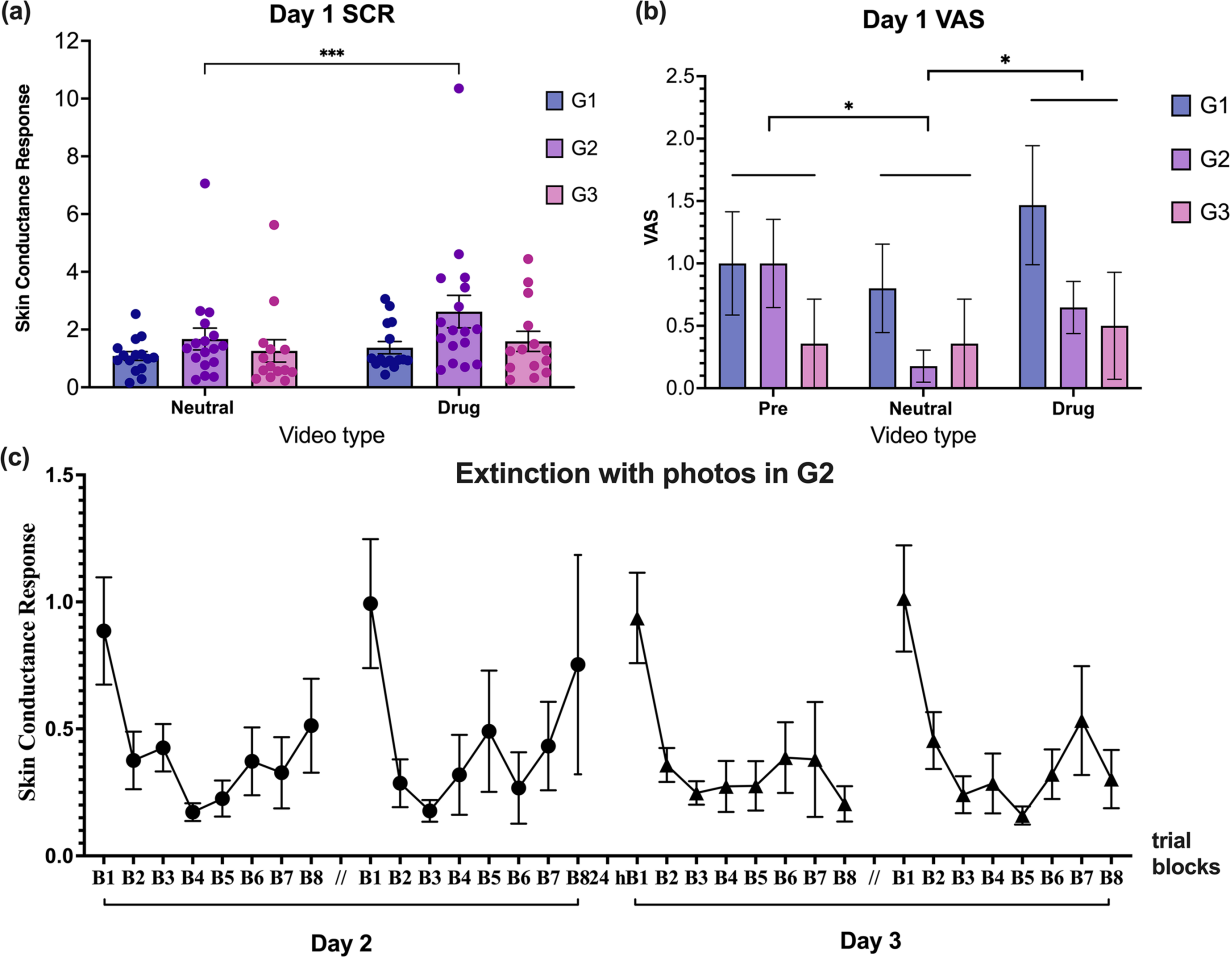
Results of Day 1 baseline testing for (a) skin conductance response and (b) subjective craving. (c) Extinction phase in G2 using drug‐related photos across trial blocks on Days 2 and 3. Each block consists of 5 photos, totalling 8 blocks across 40 trials. B, blocks. Pre, prevideo measurements; *p < 0.05, ***p < 0.001.

For SCR elicited by two types of videos, a time (1, 3 and 5 min) × video type (neutral, drug) × group (G1–G3) RMANOVA was performed with time and type as within‐subject variables and group as a between‐subject variable. The interaction effect among all three factors was significant (*F*[4, 86] = 2.53, *p* = 0.047, *η* ^2^ = 0.02), along with significant time × type interaction (*F*[2, 86] = 3.48, *p* = 0.035, *η* ^2^ = 0.01). Significant main effects were observed for video type (*F*[1, 43] = 17.27, *p* < 0.001, *η* ^2^ = 0.02) and time (*F*[2, 86] = 27.58, *p* < 0.001, *η* ^2^ = 0.06). The main effect of group was not significant. In G2, drug‐related videos elicited significantly higher SCR than neutral videos (*t* = 4.59, *p* < 0.001) (Figure 2a).

A time × group RMANOVA on participants' subjective drug craving (VAS) before and after viewing neutral and drug videos showed no significant interaction effect (*F*[4, 86] = 1.97, *p* = 0.11) but a significant main effect of time (*F*[2, 86] = 4.13, *p* = 0.019, *η* ^2^ = 0.02). The main effect of group was not significant (*F*[2, 43] = 1.22, *p* = 0.31). Post‐hoc comparisons revealed significantly higher cravings after drug videos compared to neutral videos (*t* = 2.72, *p* = 0.024, *d* = 0.31) (Figure 2b).

For systolic BP (SBP), a time × group ANOVA showed a marginally significant interaction effect (*F*[4, 86] = 2.48, *p* = 0.05, *η* ^2^ = 0.01) and a significant main effect of time (*F*[2, 86] = 4.79, *p* = 0.011, *η* ^2^ = 0.01), with no significant main effect of group. Post‐hoc comparisons showed that SBP in G1 before the experiment was higher than SBP in G1 after watching the neutral video (*t* = 3.41, *p* = 0.036, *d* = 0.48); SBP before the videos was significantly higher than after both the neutral (*t* = 2.29, *p* = 0.049, *d* = 0.19) and drug videos (*t* = 2.95, *p* = 0.012, *d* = 0.24). ANOVA results for diastolic BP (DBP) and HR showed no significant interaction or main effects (Supplementary Results SR 1b, Data S2).

Day 1 baseline testing results indicate that drug videos significantly increased participants' drug cravings and caused notable emotional changes compared to neutral videos, validating the effectiveness of the cues used.

### 3.2 Days 2 and 3 Retrieval–Intervention Analysis

#### 3.2.1 Skin Conductance Response

##### 3.2.1.1 Extinction With Photos

On Day 2, SCR data for photo stimuli were grouped into eight blocks (five trials each) and analysed using a trial (B1–B8) × phase (ext 1, ext 2) RMANOVA. There was a significant main effect of trials (*F*[7, 112] = 5.33, *p* < 0.001, *η* ^2^ = 0.16) but no significant interaction or phase effects. On Day 3, the trial effect remained significant (*F*[7, 112] = 8.83, *p* < 0.001, *η* ^2^ = 0.24), with no significant interaction or phase effects (Figure 2c).

##### 3.2.1.2 Extinction With Videos

On Day 2, SCR averaged at 1, 3, and 5 min into the video was analysed using a trial (1, 3 and 5 min) × phase (ext 1, ext 2) RMANOVA. The trial effect was significant (*F*[2, 32] = 6.18, *p* = 0.005, *η* ^2^ = 0.15), whereas interaction and phase effects were not. On Day 3, there was a marginally significant trial × phase interaction (*F*[2, 32] = 3.12, *p* = 0.058), with no significant main effects of trial or phase.

##### 3.2.1.3 Extinction With Models

On Day 2, SCR data during the first, middle and last 15 s of handling each model were analysed using a time (first, mid, end) × trial (M1–M5) × phase (ext 1, ext 2) RMANOVA. The time × trial interaction (*F*[8, 128] = 6.11, *p* < 0.001, *η* ^2^ = 0.06) and trial × phase interaction (*F*[4, 64] = 4.69, *p* = 0.002, *η* ^2^ = 0.02) were significant. Significant main effects were found for time (*F*[2, 32] = 11.83, *p* < 0.001, *η* ^2^ = 0.14) and trial (*F*[4, 64] = 12.53, *p* < 0.001, *η* ^2^ = 0.08), but not for phase. On Day 3, the time × trial interaction (*F*[8, 128] = 3.27, *p* = 0.002, *η* ^2^ = 0.04) and main effects of time (*F*[2, 32] = 13.13, *p* < 0.001, *η* ^2^ = 0.18) and trial (*F*[4, 64] = 10.05, *p* < 0.001, *η* ^2^ = 0.06) were significant. Post‐ hoc comparisons showed that SCR during the first 15 s of the first model was significantly higher than for other trials and time points.

**FIGURE 3:**
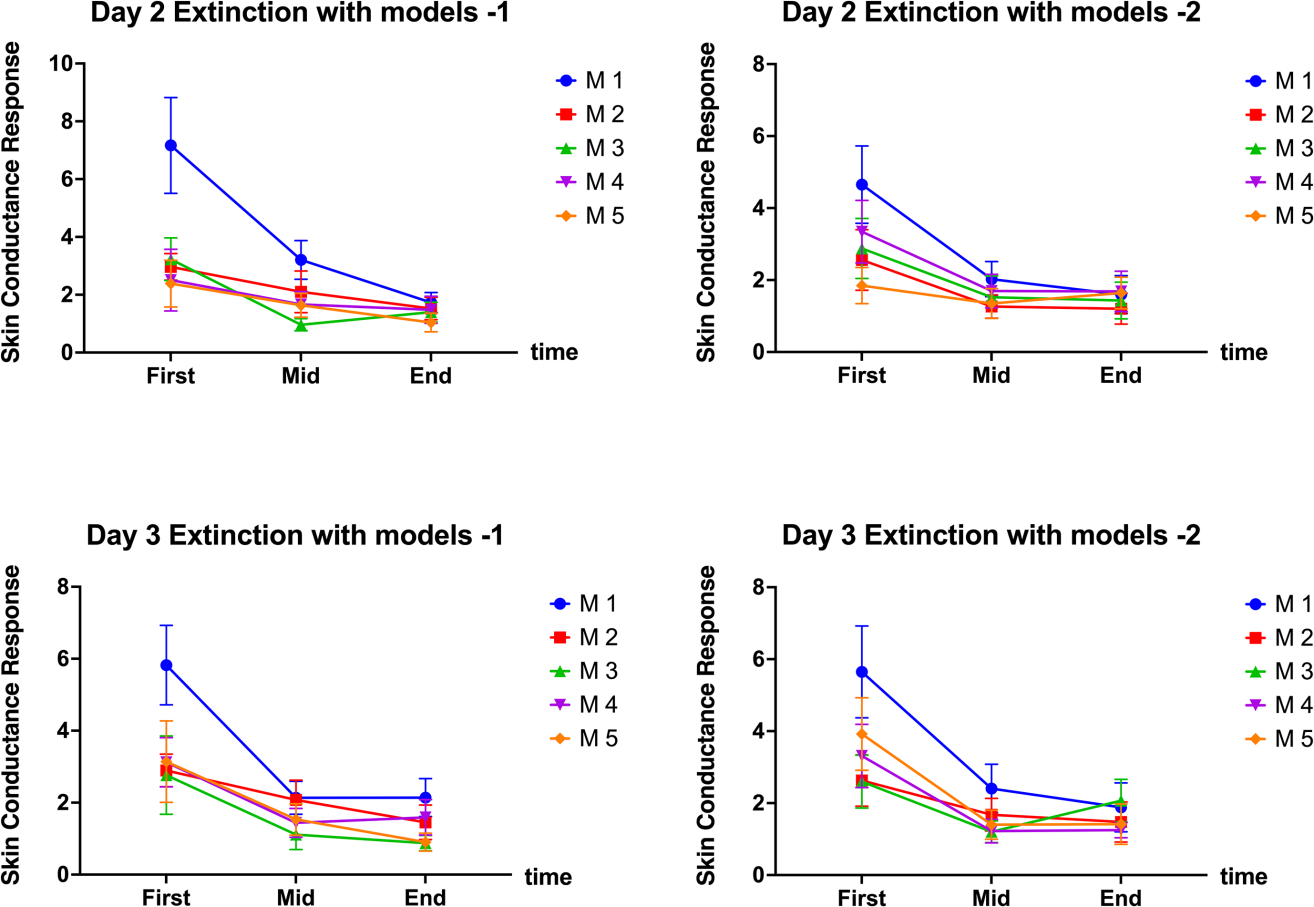
Skin conductance response during the model extinction phase on Days 2 and 3 in G2. *Note:* ‘First’, ‘mid’ and ‘end’ refer to the first 15 s, middle 15 s and last 15 s of exposure to the MA models, respectively. M1–M5 represent the first through fifth MA models.

These findings suggest that model extinction more effectively and immediately reduces emotional responses as measured by SCR, compared to photo and video extinction, with specific SCR responses to different model trials illustrated in Figure 3.

#### 3.2.2 Subjective Craving

For G2 and G3, subjective craving (VAS) levels after retrieval and interventions on Days 2 and 3 were analysed using a time (post‐retrieval, post‐intervention 1, post‐intervention 2) × group (G2, G3) RMANOVA. On Day 2, the time × group interaction was not significant (*F*[2, 58] = 2.77, *p* = 0.071), with no significant main effects for time or group. Similarly, on Day 3, there were no significant time × group interaction (*F*[2, 58] = 0.26, *p* = 0.771), and the main effects for time and group were also not significant.

These results indicate that neither extinction nor intervention on Days 2 and 3 led to significant changes in subjective drug craving, and no significant differences were observed between the groups.

#### 3.2.3 BP and HR

Analysis of SBP, DBP and HR measured at four time points on Days 2 and 3 revealed no significant differences among the groups (see SR 1–3, Data S2).

### 3.3 Day 4 Relapse Test

#### 3.3.1 Skin Conductance Response

**FIGURE 4:**
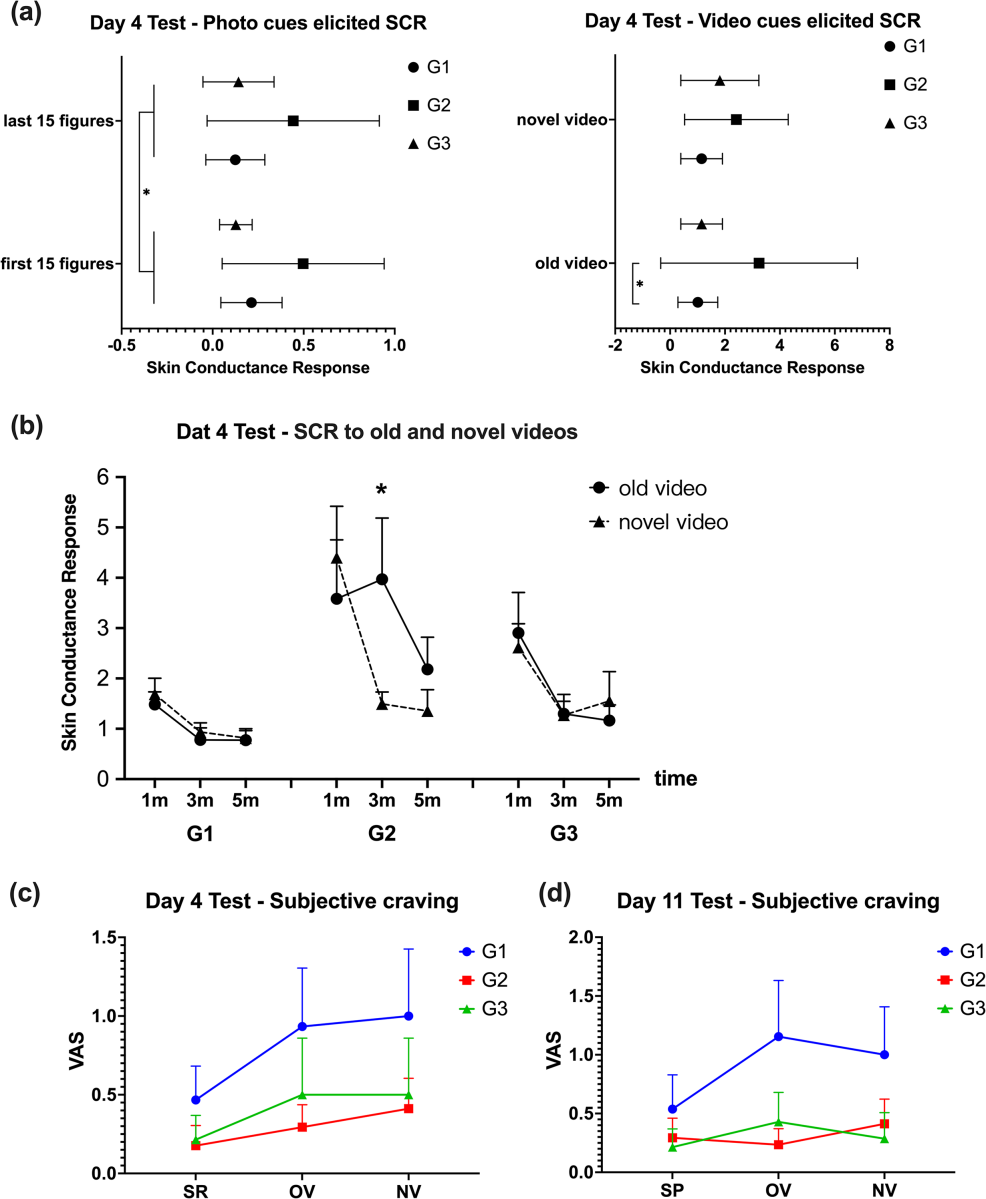
(a) Left panel: SCR induced by drug‐related photo cues on the fourth day of relapse testing. Right panel: SCR induced by drug video cues on the fourth day of relapse testing. (b) SCR changes over time in response to video cues during the Day 4 relapse test across groups. Subjective cravings were assessed on Day 4 (c) and Day 11 (d) during the relapse test and follow‐up, with measurements for spontaneous recovery (SR) without cues and video cue‐elicited VAS. Abbreviations: NV, novel video; OV, old video; SR, spontaneous recovery. *p < 0.05.

The average SCR for 30 drug‐related photos and 2 drug videos is shown in Figure 4. For the drug‐related photos, a RMANOVA with trial (first 15 photos and last 15 photos) and group as factors indicated that the interaction effect was not significant (*F*[2, 43] = 2.06, *p* = 0.14), the main effects of trial (*F*[1, 43] = 4.39, *p* = 0.042, *η* ^2^ = 0.004) and group (*F*[2, 43] = 6.08, *p* = 0.005, *η* ^2^ = 0.21) were significant. Post‐hoc analyses showed that SCR for the first 15 photos was significantly higher than for the last 15 photos (*t* = 2.10, *p* = 0.042, *d* = 0.14), and G2's SCR was significantly higher than that of G1 (*t* = 2.84, *p* = 0.014, *d* = 0.98) and G3 (*t* = 3.10, *p* = 0.01, *d* = 1.09).

A video type (old, novel) × group × time (1, 3 and 5 min) RMANOVA showed the interaction between video type, group and time was significant (*F*[4, 86] = 2.74, *p* = 0.034, *η* ^2^ = 0.02); the main effect of time was significant (*F*[2, 86] = 14.77, *p* < 0.001, *η* ^2^ = 0.06); and the main effect of group was significant (*F*[2, 43] = 4.05, *p* = 0.024, *η* ^2^ = 0.08), but the main effect of video type was not significant. This indicates that only in G2 did SCR differ between old and novel videos (with old videos showing slower SCR decrease) whereas G1 and G3 showed similar response patterns (Figure 4b).

#### 3.3.2 Subjective Craving

VAS ratings for subjective craving were measured at three time points: pretest, after Video 1 and after Video 2. A test type (spontaneous recovery, old video, novel video) × group RMANOVA showed a significant main effect of time (*F*[2, 86] = 5.15, *p* = 0.008, *η* ^2^ = 0.02) but no significant interaction or main effects for group. Post‐hoc analysis revealed that spontaneous recovery VAS was significantly lower than both old video (*t* = −2.48, *p* = 0.03, *d* = −0.27) and novel video cues (*t* = −3.00, *p* = 0.01, *d* = −0.33). Trends in VAS scores showed G1 consistently higher and G2 lower across time points (Figure 4c).

#### 3.3.3 BP and HR

The testing timepoint × group ANOVA and multiple comparisons for SBP revealed that in G2, the SBP measured before the experiment was significantly higher than after exposure to old video cues (*t* = 5.09, *p* = 0.01); the SBP recorded after photo cues was significantly higher than after old video cues (*t* = 5.23, *p* = 0.01). In contrast, the ANOVA for DBP and HR showed no significant effects for either time points or group differences. Detailed results are available in SR 1–3 Data (S2).

### 3.4 Follow‐Up Testing

#### 3.4.1 One‐Week Follow‐Up

After 1 week (Day 11), G1 lost 2 participants, whereas G2 and G3 retained all participants (*n*1 = 13, *n*2 = 17 and *n*3 = 14). For drug‐related photos, a trial (first 15 photos and last 15 photos) × group RMANOVA revealed no interaction but a significant main effect for the group (*F*[2, 41] = 7.59, *p* = 0.002, *η* ^2^ = 0.24), with G2 showing significantly higher SCR than G3 (*t* = 3.89, *p* = 0.001, *d* = 1.30) (Figure 6a). A video type (old, novel) × group × time analysis found significant time effects (*F*[2, 82] = 16.82, *p* < 0.001, *η* ^2^ = 0.08) but no significant group or video type effects, suggesting diminished SCR differences between novel and old videos after 1 week, though SCR levels at the first minute remained significantly higher. VAS scores indicated a trend for higher cravings in G1 compared to G2 for old video cues (*F*[2, 41] = 2.60, *p* = 0.087, *η* ^2^ = 0.11; *t* = 2.21, *p* = 0.08, *d* = 0.81) (Figure 6b). No significant differences were found for spontaneous recovery or novel video cues. For BP and HR, in G1, SBP was significantly higher before the experiment than after exposure to old video cues (*t* = 5.56, *p* = 0.009). Furthermore, in G1, DBP after photo cues was significantly lower than after exposure to both old (*t* = 4.97, *p* = 0.02) and novel video cues (*t* = 4.86; *p* = 0.02).

#### 3.4.2 One‐Month Follow‐Up

**FIGURE 5:**
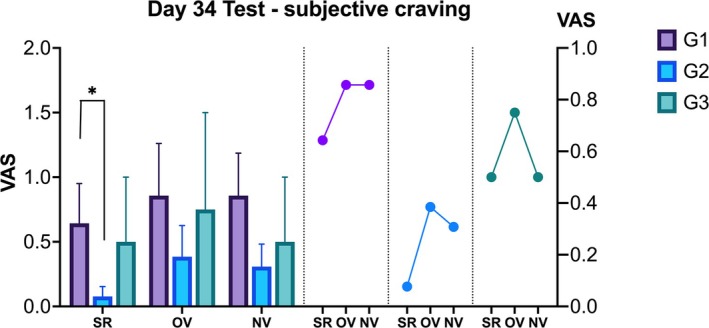
Subjective craving at a 1‐month follow‐up. During spontaneous recovery without cues, G1 showed significantly higher VAS scores than G2. No significant differences were observed in VAS scores triggered by old or novel videos. Abbreviations: NV, novel video; OV, old video; SR, spontaneous recovery. *p < 0.05.

A month later, a relapse test was conducted. Due to a severe COVID‐19 outbreak, G3 saw a significant participant loss (around 70%), leaving *n*1 = 13, *n*2 = 13 and *n*3 = 4. SCRs for photos and videos across groups were similar. ANOVA revealed no significant group effects for old or novel videos, indicating that SCR had become insensitive as a measure of MUD memory retention. Due to G3's small sample size, only G1 and G2 were compared. Independent samples *t* tests showed that G1 had significantly higher VAS scores than G2 during spontaneous recovery without cues (*t* = 1.73, *p* = 0.048, *d* = 0.66, *power* [1−*β*] = 0.50) (Figure 5), but no significant differences were found for VAS scores triggered by old or novel videos. To test the effect of excluding G3 data on the research results, sensitivity analysis was performed. Comparing the results of time × group RMANOVA when G3 data were included and excluded, it was found that there was no significant change in the results (interaction effect *F*[4, 56] = 0.107, *p* = 0.979; *F*[2, 50] = 0.046, *p* = 0.955), indicating that excluding G3 did not affect overall research conclusions. Additionally, in G1, the DBP before the experiment was significantly higher than after photos (*p* = 0.03), and in G2, the DBP before the experiment was significantly lower than after old video cues (*p* = 0.008) (SR 1–3, Data S2).

### 3.5 Full Process Analysis: Time‐Course Changes

#### 3.5.1 Physiological Indicators

**FIGURE 6:**
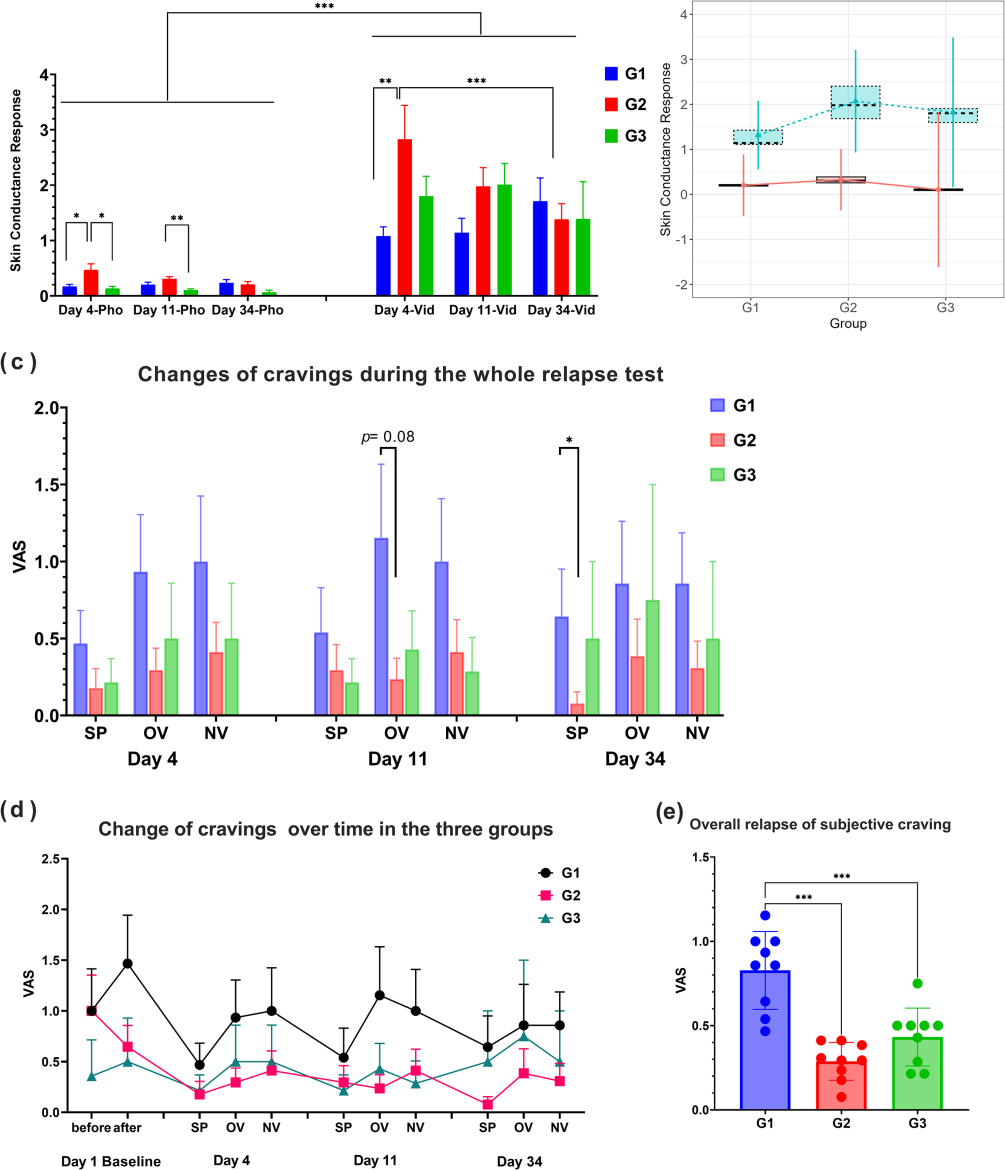
Physiological (SCR) and psychological (VAS) indicators in each group on Days 4, 11 and 34. (a) Time course of SCR changes in response to drug cues throughout the relapse tests in each group. (b) Mixed linear model (MLM) analysis results, with group and cue type (photo and video) as fixed effects and tracking time as a random effect. (c) Time course of craving across different test types during the relapse tests in each group. (d) The change of subjective cravings from baseline to tests in G1–G3. (e) Overall relapse of subjective craving during the test period in each group. Abbreviations: NV, novel video; OV, old video; SR, spontaneous recovery. *p < 0.05, ***p < 0.001.

The SCR data collected on Days 4, 11 and 34 were analysed using a time × cue type (photo, video) × group ANOVA. The analysis revealed a significant three‐way interaction effect (*F*[4, 54] = 3.05, *p* = 0.024, *η* ^2^ = 0.02), a significant time × group interaction effect (*F*[4, 54] = 3.78, *p* = 0.009, *η* ^2^ = 0.04), a significant main effect of cue type (*F*[1, 27] = 50.41, *p* < 0.001, *η* ^2^ = 0.27) and a marginally significant main effect of time (*F*[2, 54] = 3.15, *p* = 0.051, *η* ^2^ = 0.02). Post‐hoc comparisons indicated that SCR elicited by video cues was significantly higher than that by photo cues. Furthermore, G2's SCR in the initial relapse test was significantly higher than that of G1 and significantly decreased by the 1‐month test (Figure 6a).

Given the missing data at follow‐up points, a linear mixed model (LMM) was employed to assess effects and control the impact of missing data on the results. Group and cue type (photo and video) were treated as fixed effects, whereas tracking time was treated as a random effect. The maximum likelihood method was used for model fitting. The fixed effect of cue type was significant (*p* < 0.001), whereas the fixed effect of group and the interaction effect were not significant (Figure 6b).

#### 3.5.2 Psychological Indicators

To assess the effect of the intervention on subjective cravings over time, we conducted a RMANOVA comparing the VAS scores from the baseline (Day 1) to the subsequent test days (Days 4, 11 and 34). The analysis followed a 4 × 2 × 3 design (time: Days 1, 4, 11 and 34; test type: spontaneous recovery, post‐video; group: G1–G3). For the analysis of old video cues, a significant main effect of time was observed (*F*[3, 81] = 4.59, *p* = 0.005, *η* ^2^ = 0.02) but no significant interactions or main effects for other factors. Post‐hoc tests showed a trend toward higher cravings at the Day 1 baseline compared to Day 4 (*t* = 2.27, *p* = 0.126), Day 11 (*t* = 2.54, *p* = 0.098) and Day 34 (*t* = 2.56, *p* = 0.098). For novel video cues, a significant main effect of time was also found (*F*[3, 81] = 5.59, *p* = 0.002, *η* ^2^ = 0.02). Post‐hoc comparisons revealed that cravings were marginally higher on Day 1 compared to Day 34 (*t* = 2.79, *p* = 0.058, *d* = 0.39). Changes in subjective cravings from baseline to the test days for each group (G1–G3) are shown in Figure 6d.

In the inner‐group RMANOVA, the analysis of time × test type (spontaneous recovery, post‐old video) indicated a significant main effect of time for G2 (*F*[3, 36] = 3.55, *p* = 0.024, *η* ^2^ = 0.09) but not for G1 (*F*[3, 36] = 0.23, *p* = 0.88). A similar pattern was observed in the RMANOVA for time × test type (spontaneous recovery, post‐novel video), with a significant main effect for G2 (*F*[3, 36] = 3.80, *p* = 0.018, *η* ^2^ = 0.08) but not for G1 (*F*[3, 36] = 0.45, *p* = 0.72) (Figure 6d).

One‐way ANOVA analyses between groups for each test type revealed a significant difference in spontaneous recovery between G1 and G2 1 month later (*p* = 0.048) and a trend toward significance for old video cues between G1 and G2 1 week later (*p* = 0.08) (Figure 6c).

These findings suggest that R‐E led to a significant decrease in cravings, whereas the R group did not show a significant change over time. In contrast to the SCR findings, the intergroup relationships did not significantly change across the three tracking times for cravings.

#### 3.5.3 Overall Craving Trends During the Test Period

To explore overall craving trends across groups throughout the entire testing and tracking period, the average VAS scores from Days 4, 11 and 34 were analysed using a one‐way ANOVA. A significant group effect was observed (*F*[2, 24] = 22.10, *p* < 0.001, *η* ^2^ = 0.65). Post‐hoc comparisons revealed that cravings in G1 were significantly higher than in G2 (*t* = 6.42, *p* < 0.001, *d* = 3.03) and G3 (*t* = 4.70, *p* < 0.001, *d* = 2.22), with no significant difference between G2 and G3 (*t* = 1.72, *p* = 0.219). These findings provide supplementary evidence suggesting that the R group exhibited the strongest MUD‐related memory, whereas cravings in the R‐E and R‐CT groups were significantly reduced (Figure 6d).

## 4 Discussion

The investigation found that G2 (retrieval–extinction, R‐E) effectively reduced drug cravings in the subjects, with cravings in the spontaneous recovery condition (without cues) 1 month later being significantly lower than those in G1 (retrieval–no intervention, R). The G3 (retrieval–cognitive task, R‐CT) also showed benefits in inhibiting cravings in the follow‐up tests. Specifically, R‐E led to a significant decrease in cravings, whereas the R group did not exhibit significant changes over time. Overall, cravings during the test period were significantly suppressed in both the R‐E and R‐CT groups compared to the R group. These findings suggest that the retrieval–extinction paradigm can effectively inhibit the recurrence of addictive memories in MUD patients, consistent with previous studies [22, 23]. In terms of intervention methods, different behavioural interventions have been shown to be effective in disrupting addictive memories in other types of substances. For example, in alcohol addiction, interventions such as counterconditioning [13], working memory training [17] and cognitive reappraisal [18] were utilized following memory retrieval. This study is the first to investigate the retrieval–cognitive intervention method for MUD memory erasure, supporting the feasibility of post‐retrieval interventions, which is hypothesized to utilize memory reconsolidation principles, and demonstrating the potential for applying various behavioural strategies within this framework.

Based on the current results, we cannot draw firm conclusions regarding the relative superiority of the R‐E method over the R‐CT method, especially considering the significant attrition of participants in the R‐CT group during the 1‐month follow‐up. We hypothesize that the underlying mechanisms of both methods are similar, as both rely on memory reconsolidation—a process where reactivated memories become unstable, allowing for potential modification or disruption within a limited time window, typically up to 6 h. However, due to the absence of a no‐retrieval control group, we cannot confirm this hypothesis. Although the underlying mechanisms remain unproven, both strategies have demonstrated efficacy, which holds important clinical implications for the treatment of MUDs. In particular, R‐CT offers a novel and practical way to interfere with drug‐related memories through the use of Tetris following memory reactivation. Although behavioural interventions are flexible, the qualitative experiences of participants in completing these interventions vary. For example, playing Tetris may be less stressful than extinction and simpler to implement. Hence, although both R‐E and R‐CT showed comparable efficacy in reducing cravings, R‐CT (e.g., playing Tetris) may be more suitable for clinical use due to its ease of implementation and better patient acceptability.

This study observed notable differences between the physiological indicator (SCR) and the psychological indicator (VAS) in measuring MUD relapse. First, extinction training immediately affected SCR on the Days 2 and 3 but had no immediate effect on VAS. Second, during the relapse test on Day 4 and 1 week later, SCR and VAS displayed opposite trends: G2 had the highest SCR and the lowest VAS among the groups. This suggests that SCR and VAS differ in sensitivity and scope when measuring MUD severity. SCR, which reacts quickly but is short‐lived and time sensitive, is effective for immediate and short‐term effects but less suitable for long‐term tracking. In contrast, VAS is more stable, less influenced by time and better suited for long‐term tracking, though its immediate effects are less pronounced, typically emerging after 24 h. Although it is true that craving can change over time, previous research has shown that self‐reported measures like VAS provide a more robust and stable assessment of long‐term emotional states or cravings [31, 32]. Additionally, the divergence between SCR and VAS in short‐term relapse suggests that physiological and psychological indicators might not be fully aligned when assessing the elimination of addictive memories. This aligns with previous findings by Tolliver et al. [4], who reported that although exposure to methamphetamine‐related cues can trigger both cravings and autonomic responses, physiological reactions like HR and SCR are poorly correlated with subjective craving changes and do not fully capture the craving state.

The study found no significant differences in psychological indicators between relapse triggered by novel versus old cues in MUD memory. Regarding physiological indicators, only G2 showed a difference between novel and old videos during the initial test on Day 4, with no differences observed in the other groups or subsequent relapse tests. This suggests that the inhibitory effects of R‐E or R‐CT on MUD memory may generalize to novel cues, potentially preventing relapse triggered by new MA cues. This finding is consistent with previous smoking studies, where Germeroth et al. [14] reported that R‐E or R‐CT training significantly reduced cravings and daily smoking triggered by both familiar and novel smoking cues. However, further research is needed to confirm whether the inhibitory effects of this paradigm can be robustly generalized to novel cues.

In this study, cue‐induced craving was used to assess the strength and relapse potential of MA‐related memories. Although the use of drug‐related cues can elicit temporary discomfort or increased craving, it is a scientifically validated method necessary for understanding possible mechanisms and evaluating intervention efficacy. To mitigate risks, several precautions were taken: (1) Participants were informed about craving induction and could withdraw at any time; (2) only brief, noninvasive cue exposure was used; (3) emotional support was provided after sessions; and (4) trained staff monitored participants' well‐being. Importantly, participation did not affect treatment outcomes or length of stay in the rehabilitation centre. These procedures were approved by the ethics committee and followed ethical standards for vulnerable populations.

This study has several limitations. First, the small sample size, which was predominantly male, limits both the representativeness of the sample and the statistical power of the findings. Additionally, significant attrition in G3 during the 1‐month follow‐up due to force majeure hindered a thorough evaluation of the long‐term effects of retrieval interventions, particularly cognitive interventions, on MUD‐related relapse. Second, the lack of a control group specifically assessing the effect of retrieval–extinction (retrieval dependence) and the absence of craving measurements for photo cues in the relapse test further limit the interpretation of the results. Third, considering the differences between a detox centre and a natural environment is crucial. Specifically, participants in the detox centre were unable to be assessed for actual drug consumption as an outcome, as access to drugs was restricted. In a natural setting, participants might have the opportunity to access drugs, which could provide a more accurate measure of relapse or treatment efficacy based on actual consumption. Furthermore, the knowledge that drugs were unavailable in the detox centre may have reduced cue reactivity. Finally, the 1‐month follow‐up period is relatively short for evaluating the long‐term efficacy of the interventions on MUD relapse, and future studies should incorporate longer follow‐up periods to assess sustained effects.

## 5 Conclusions

Collectively, our findings suggest that both retrieval–extinction and retrieval–cognitive task interventions offer advantages over retrieval alone in treating MUD. These results support the potential of noninvasive, behavioural post‐retrieval strategies for targeting drug‐related memories in real‐world settings, such as compulsory rehabilitation centres. The observed weak correlation between physiological and psychological relapse indicators highlights the need for multimodal assessment tools in addiction research and clinical practice. This study offers new strategies for MUD intervention and valuable insights for clinical treatment.

## Author Contributions

**Junjiao Li:** conceptualization, methodology, software, validation, formal analysis, data curation, writing – original draft, writing – review and editing, visualization, funding acquisition. **Yuanyuan Dong:** conceptualization, methodology, software, investigation, data curation. **Wei Chen:** methodology, software, validation, data curation, writing – review and editing, funding acquisition. **Wang Jian:** software, investigation, resources. **Xifu Zheng:** conceptualization, supervision, project administration, funding acquisition.

## Ethics Statement

The study was approved by the Ethics Committee at South China Normal University (Approval Number: SCNU‐PSY‐2021‐329).

## Conflicts of Interest

The authors declare no conflicts of interest.

## Supporting information


**Data S1.** Supporting Information.

**Data S2.** Supporting Information.

## Data Availability

The data that support the findings of this study are available on request from the corresponding author. The data are not publicly available due to privacy or ethical restrictions.
