# Supplementary material for: Exploring Post‐Retrieval Strategies to Reduce Drug Craving in Methamphetamine Use Disorders
Source: Addict Biol. 2025 Jun 19;30(6):e70049. doi: 10.1111/adb.70049 (PMC12178206; doi:10.1111/adb.70049)
Supplement: Supplementary file 1 — Data S1. Supporting Information. [file ADB-30-e70049-s002.pdf]

Supplementary Materials

SM 1 Example of experimental materials

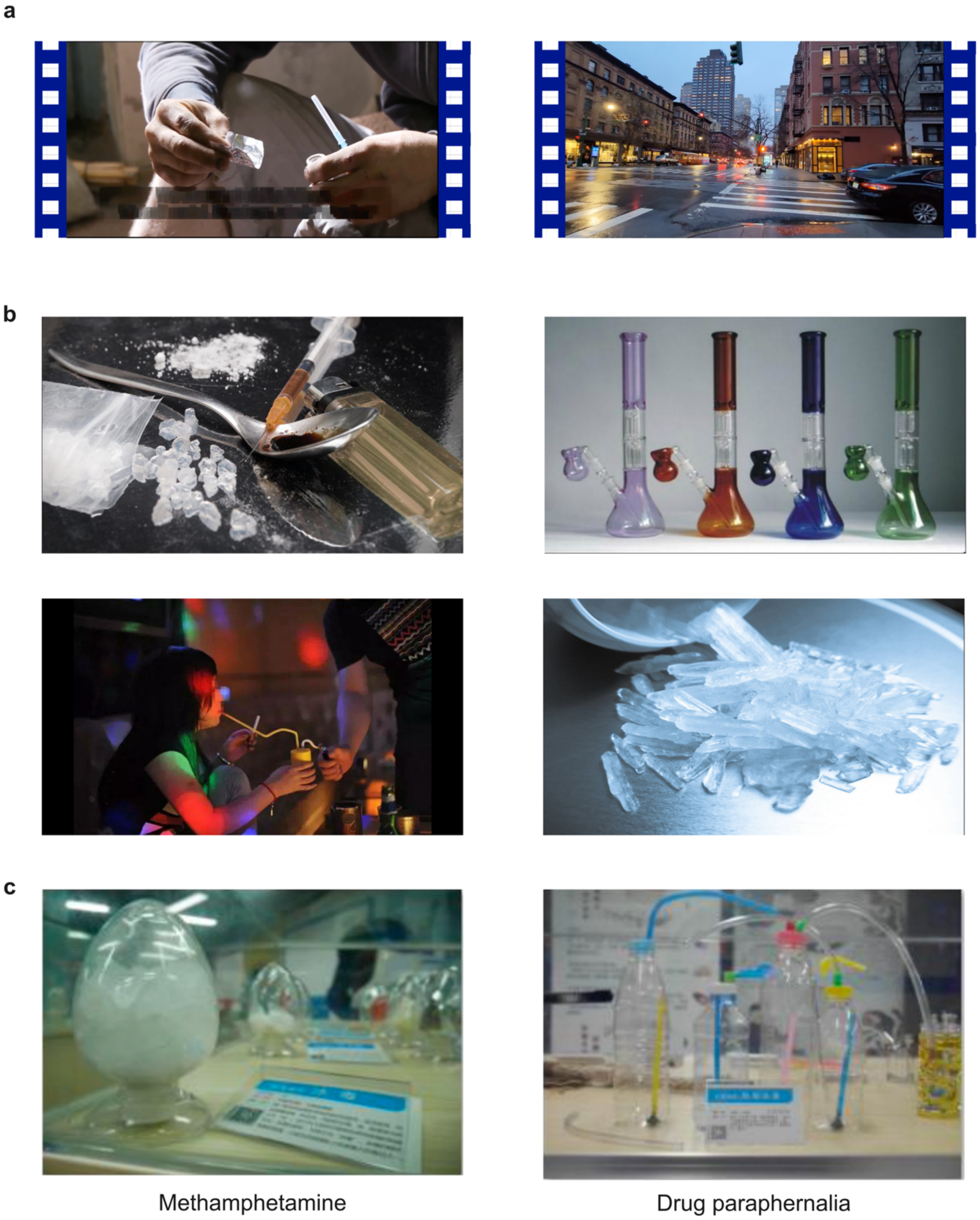

SM 1 Example of experimental materials. a: Video materials (left: drug; right: neutral); b: Photo materials; c: Model materials, including drug paraphernalia and drug models, provided by the drug rehabilitation education base.

SM 2 Experimental materials: methamphetamine photo (partial view).

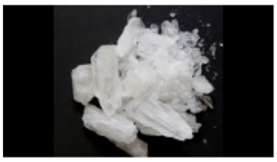
1-10

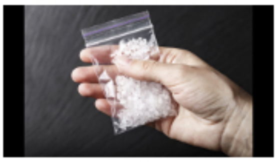
1-11

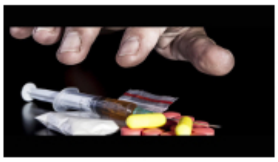
1-12

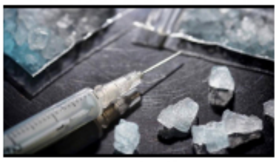
1-14

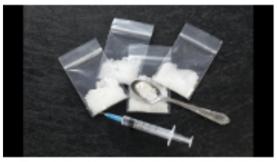
1-21

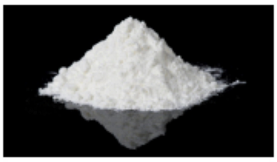
1-22

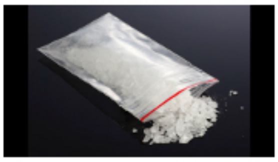
1-23

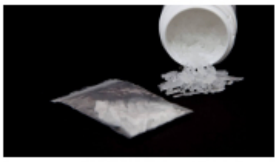
1-24

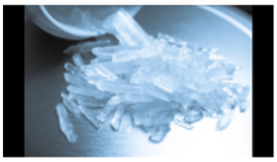
1-25

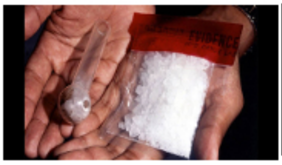
1-27

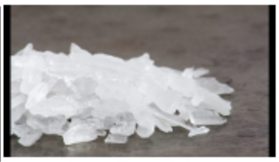
1-28

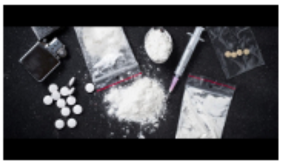
1-29

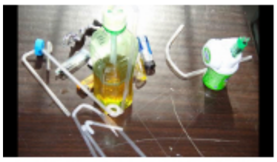
1-38

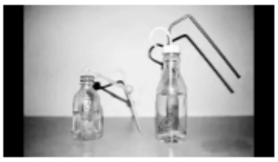
1-39

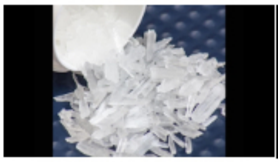
1-40

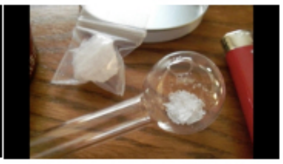
1-42

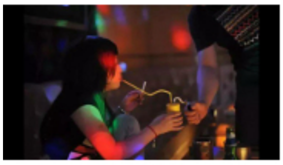
2-1

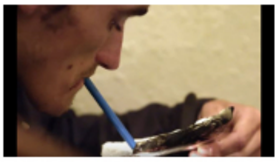
2-6

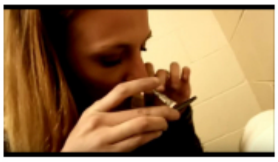
2-7

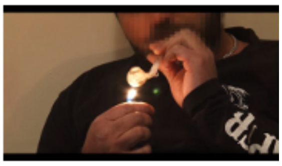
2-8

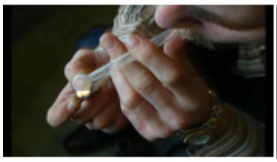
2-9

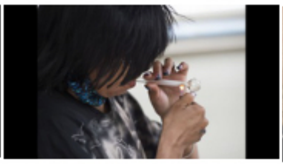
2-11

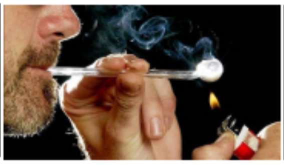
2-13

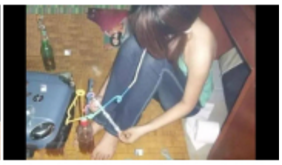
2-40

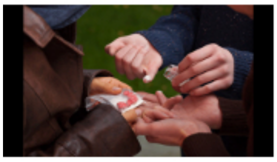
2-44

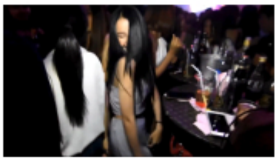
3-1

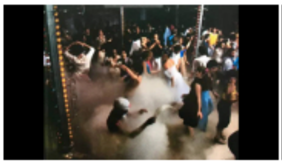
3-2

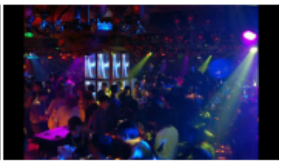
3-3

SM 3 Appraisal data of the experimental materials

Data of material appraisal

Material number	Arousal	Pleasure	Craving
Photos			
1-1	2.1	2.4	1.9
1-2	1.6	1.2	1.4
1-3	1.7	1.6	1.5
1-4	1.6	1.7	1.8
1-5	1.9	2.1	2.1
1-6	1.2	1.4	1.2
1-7	1.2	1.5	1.4
1-8	1.8	2	1.4
1-9	1.8	1.7	1.6
1-10	1.2	1.1	1.1
1-11	1.9	1.9	1.9
1-12	1.3	1.7	1.5
1-13	1.8	1.9	1.8
1-14	1.3	1.4	1.7
1-15	1.6	1.5	1.5
1-16	2.1	2	1.8
1-17	2.2	2.3	2.1
1-18	2.4	2.4	2.2
1-19	1.5	2	1.7
1-20	2.5	2.6	2.6
1-21	2.1	2.2	2
1-22	1.7	1.4	1.2
1-23	1.9	1.5	1.3
1-24	2.2	2.5	2.2
1-25	2	2	2
1-26	2.5	2.6	2.5
1-27	2.1	2	1.7
1-28	1.7	2	1.8
1-29	2.1	2.3	1.7
1-30	2.4	2.4	1.9
2-1	1.7	1.4	1.2
2-2	1.9	1.5	1.3
2-3	2.2	2.7	1.9
2-4	1.6	1.1	1.3
2-5	2.3	1.7	1.6

2-6	1.4	1.6	0.8
2-7	1.7	1.7	1.6
2-8	1.2	1.4	1.1
2-9	1.7	1.6	1.6
2-10	1.6	1.6	1.2
2-11	1.8	1.5	1.5
2-12	1.8	1.8	1.5
2-13	2.4	2.0	1.8
2-14	1.6	1.1	1.2
2-15	2.1	1.7	1.5
2-16	1.7	1.3	1.4
2-17	1.6	1.4	1.2
2-18	2.2	1.9	1.8
2-19	2.2	2.1	1.8
2-20	1.9	1.6	1.4
2-21	1.6	1.3	1.1
2-22	1.9	2.0	1.5
2-23	2.3	2.5	2.1
2-24	2.0	1.9	1.5
2-25	2.1	1.8	1.2
2-26	2.7	2.3	2.1
2-27	2.5	1.9	2.0
2-28	2.2	2.5	1.6
2-29	2.5	2.1	2.0
3-1	2.0	1.6	1.6
3-2	2.2	1.9	1.8
3-3	2.3	2.1	1.6
3-4	2.6	1.4	1.3
3-5	1.9	1.5	1.5
3-6	2.2	1.2	1.5
3-7	2.7	3.3	2.8
3-8	3.5	3.7	2.8
3-9	3.3	3.7	3.2
3-10	3.7	3.8	3.1
Videos			
1	2.10	2.11	1.46
2	1.38	1.76	1.40
3	1.85	1.47	1.30
4	1.87	2.03	1.03
5	2.13	2.41	1.72
6	2.07	2.28	1.34
