# Supplementary material for: Exploring Post‐Retrieval Strategies to Reduce Drug Craving in Methamphetamine Use Disorders
Source: Addict Biol. 2025 Jun 19;30(6):e70049. doi: 10.1111/adb.70049 (PMC12178206; doi:10.1111/adb.70049)
Supplement: Supplementary file 2 — Data S2. Supporting Information. [file ADB-30-e70049-s001.pdf]

Supplementary Results

SR 1 Blood pressure: systolic blood pressure (SBP)

SR1 a. Retrieval-extinction phase: Day 2 & 3

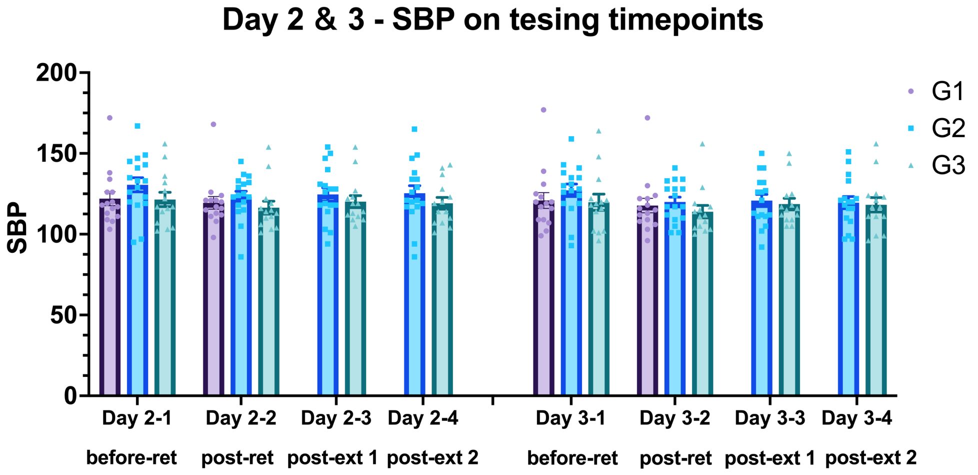

SR1 a. Systolic blood pressure at each testing timepoint during Day 2 and Day 3 retrieval and extinction phases. **before-ret**: before memory retrieval; **post-ret**: after memory retrieval; **post-ext 1**: after the first extinction training; **post-ext 2**: after the second extinction training.

SR1 b. Baseline and Testing phase

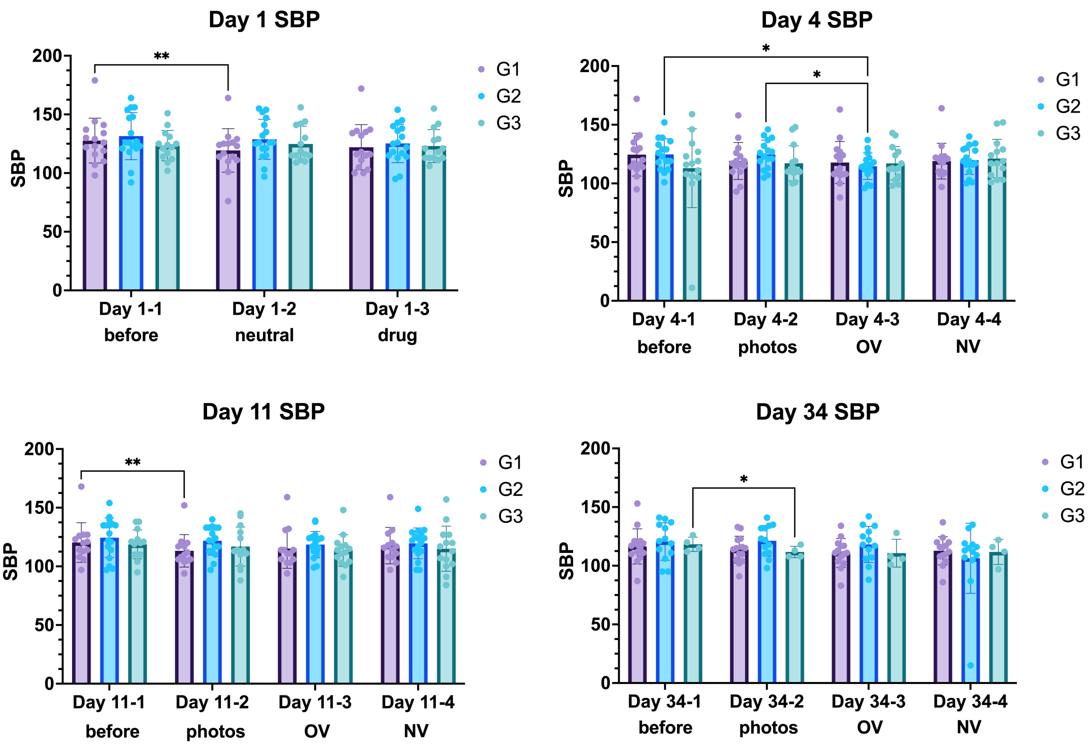

SR1 b. Systolic blood pressure at each testing timepoint during the baseline test on Day 1, relapse testing on Day 4, and follow-ups on Days 11 and 34. **OV**: old video; **NV**: novel video.

SR 2 Blood pressure: diastolic blood pressure (DBP)

SR 2 a. Retrieval-extinction phase: Day 2 & 3

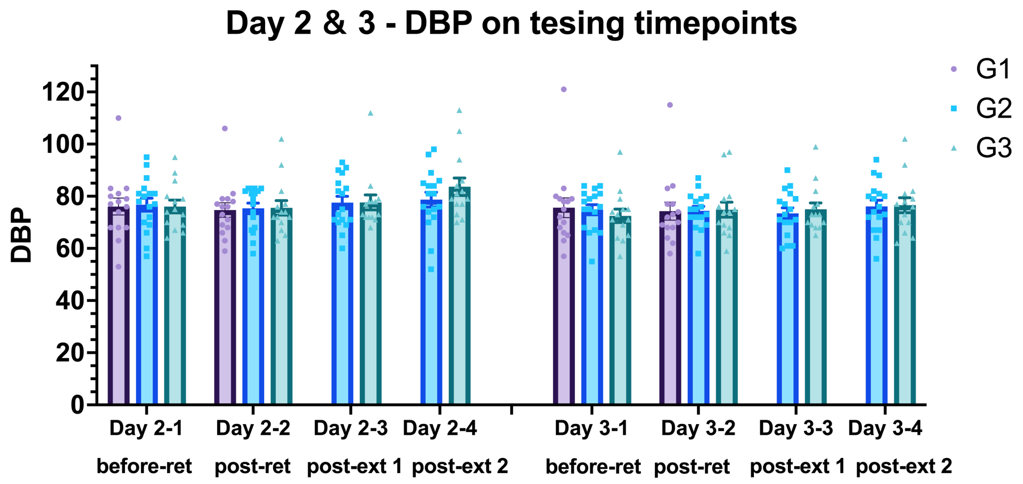

SR 2 a. Diastolic blood pressure at each testing timepoint during Day 2 and Day 3 retrieval and extinction phases. **before-ret**: before memory retrieval; **post-ret**: after memory retrieval; **post-ext 1**: after the first extinction training; **post-ext 2**: after the second extinction training.

SR 2 b. Baseline and Testing phase

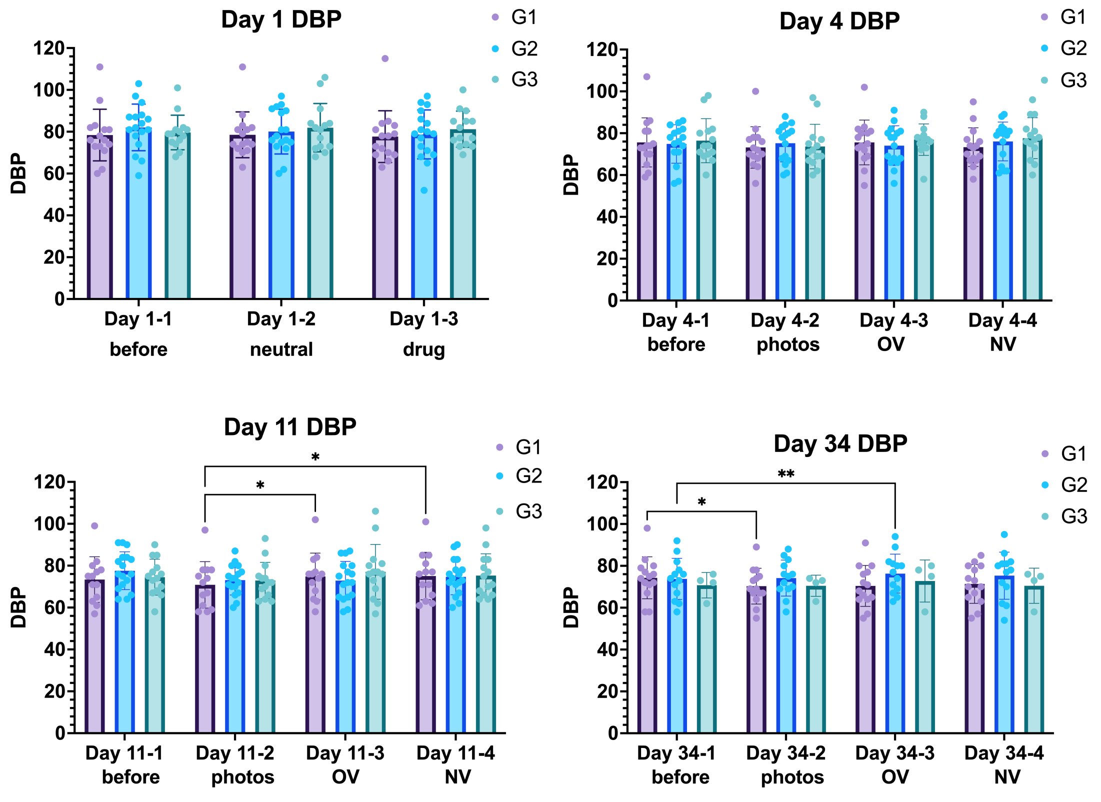

SR 2 b. Diastolic blood pressure at each testing timepoint during the baseline test on Day 1, relapse testing on Day 4, and follow-ups on Days 11 and 34. **OV**: old video; **NV**: novel video.

SR 3 Heart Rate

SR 3 a. Retrieval-extinction phase: Day 2 & 3

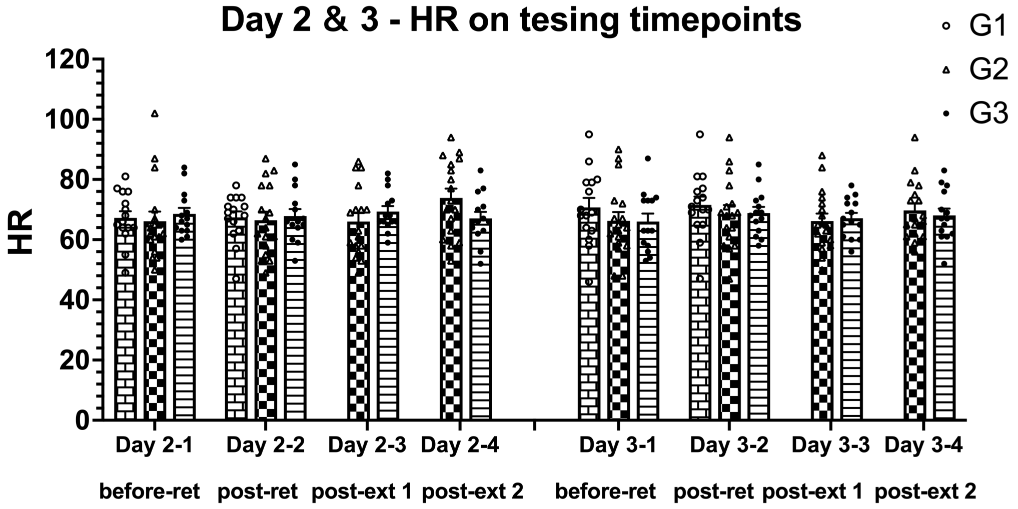

SR 3 a. Heart rate at each testing timepoint during Day 2 and Day 3 retrieval and extinction phases. **before-ret**: before memory retrieval; **post-ret**: after memory retrieval; **post-ext 1**: after the first extinction training; **post-ext 2**: after the second extinction training.

SR 3 b. Baseline and Testing phase

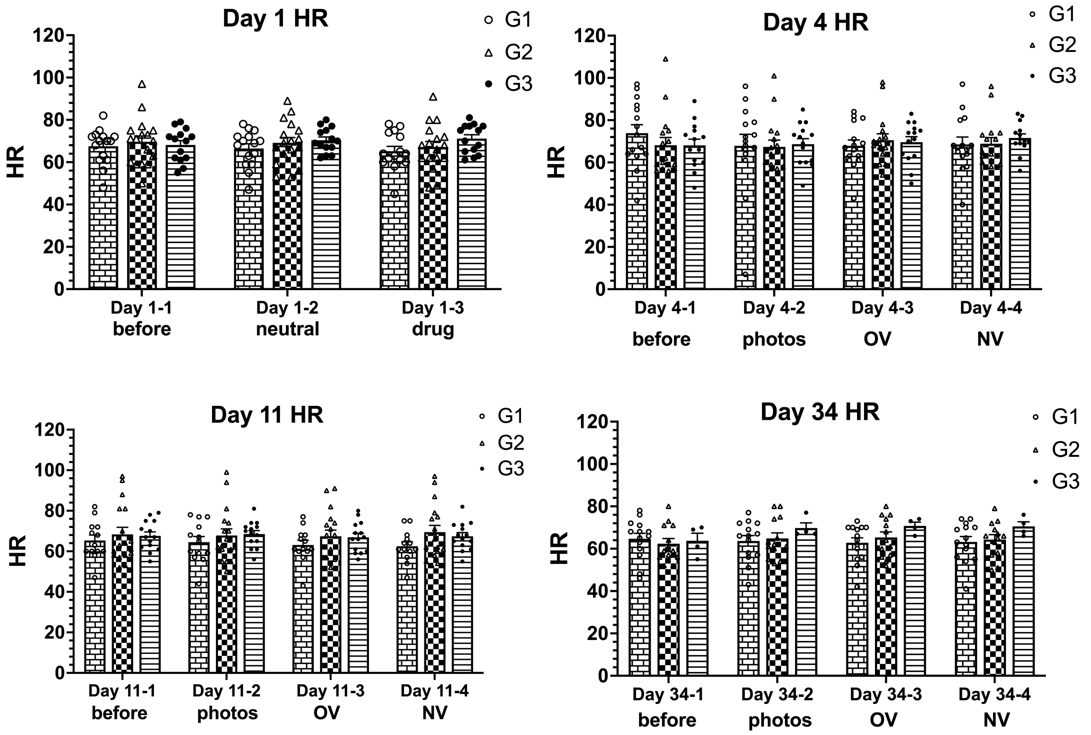

SR 3 b. Heart rate at each testing timepoint during the baseline test on Day 1, relapse testing on Day 4, and follow-ups on Days 11 and 34. **OV**: old video; **NV**: novel video.
